# Late-onset esophagogastric anastomotic fistula managed without continuous fasting: a case report

**DOI:** 10.1093/jscr/rjaf796

**Published:** 2025-10-06

**Authors:** Yuhui Gong, Yu Zhang, Haitao Ma, Wei Jiang, Jiangjiang Liu, Yunteng Kang, Jialiang Liu, Xiaojun Yu

**Affiliations:** Department of Thoracic Surgery, The Fourth Affiliated Hospital of Soochow University, No. 9, Chongwen Road, Suzhou, Jiangsu, 21500, China; Department of Thoracic Surgery, The Fourth Affiliated Hospital of Soochow University, No. 9, Chongwen Road, Suzhou, Jiangsu, 21500, China; Department of Thoracic Surgery, The Fourth Affiliated Hospital of Soochow University, No. 9, Chongwen Road, Suzhou, Jiangsu, 21500, China; Department of Thoracic Surgery, The Fourth Affiliated Hospital of Soochow University, No. 9, Chongwen Road, Suzhou, Jiangsu, 21500, China; Department of Thoracic Surgery, The Fourth Affiliated Hospital of Soochow University, No. 9, Chongwen Road, Suzhou, Jiangsu, 21500, China; Department of Thoracic Surgery, The Fourth Affiliated Hospital of Soochow University, No. 9, Chongwen Road, Suzhou, Jiangsu, 21500, China; Department of Thoracic Surgery, The Fourth Affiliated Hospital of Soochow University, No. 9, Chongwen Road, Suzhou, Jiangsu, 21500, China; Department of Thoracic Surgery, The Fourth Affiliated Hospital of Soochow University, No. 9, Chongwen Road, Suzhou, Jiangsu, 21500, China

**Keywords:** case report, esophagogastric anastomotic fistula, noncontinuous fasting management

## Abstract

Esophagogastric anastomotic fistula is a common complication following esophageal cancer surgery, typically occurring within the first postoperative week. Conventional management requires prolonged fasting until complete fistula closure, which significantly impacts patient quality of life. We present a case of a male who developed an esophagogastric anastomotic fistula 2 months postoperatively, complicated by a concurrent gastrobronchial fistula. Endoscopic evaluation revealed persistent gastric wall defects at the fistula site. As the disease progressed, thickened visceral pleura formed dense adhesions with the damaged gastric tissue, effectively sealing the defect and preventing digestive fluid leakage into the thoracic cavity. By promptly promoting gastric emptying and reducing gastric acid secretion, the patient resumed oral intake without developing severe infections or complications.

## Introduction

Postesophagectomy anastomotic fistula occurs in ~10% of cases, most commonly during the early postoperative period and diagnosed via endoscopy or upper gastrointestinal imaging. However, fistulas developing more than 2 months after surgery are rare, and their etiology remains unclear. Current management strategies include fasting, drainage, nutritional support, endoscopic closure, interventional closure, and surgical revision. While treatment outcomes have improved significantly [1], the prolonged recovery time reduced quality of life and the mortality rate remained at 30%. Treatment plans for postesophagectomy fistulas should be individualized based on fistula characteristics (location, size) and patient comorbidities. Conservative approaches combined with endoscopic techniques are now standard, though surgical intervention remains necessary for complex cases [2].

## Case report

**Figure 1:**
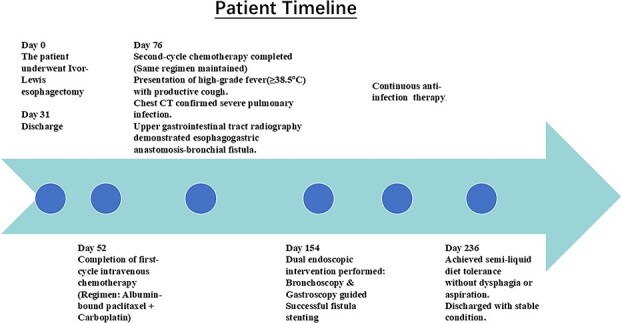
Timeline of event.

**Figure 2:**
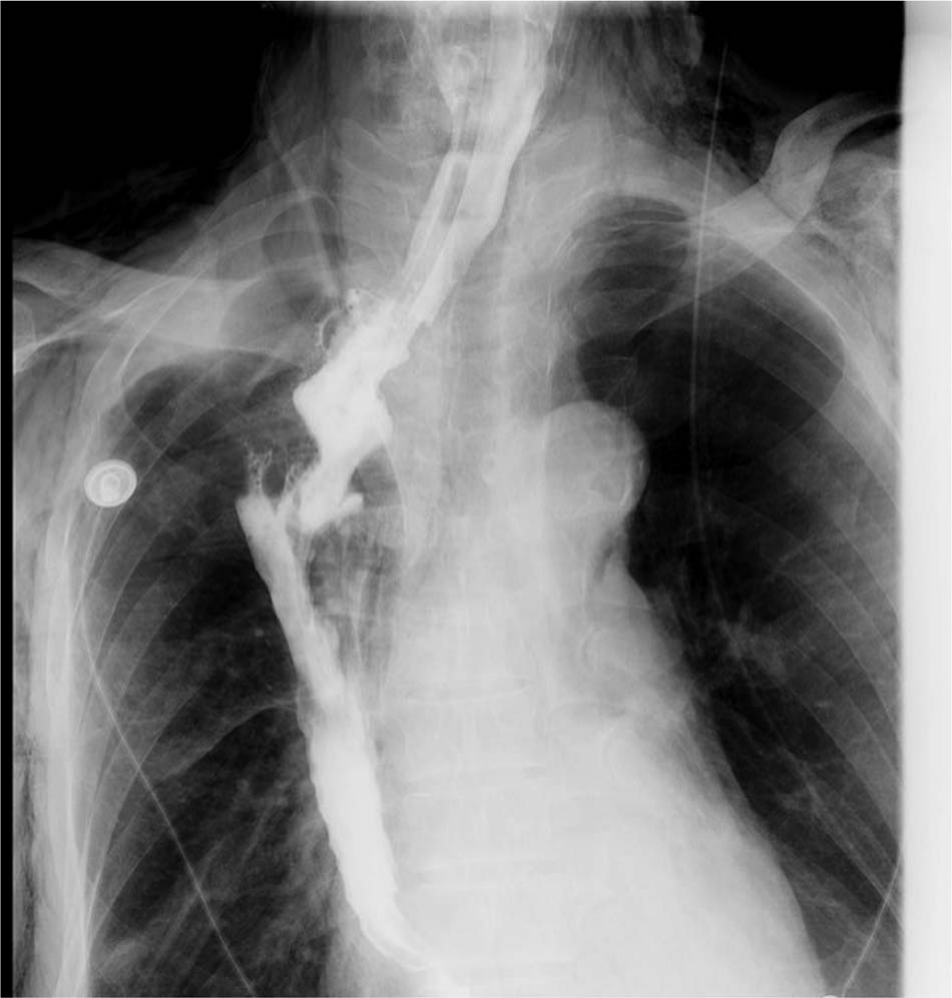
Upper gastrointestinal series performed during postoperative week 1.

A concise illustration of the disease progression is presented in Fig. 1. A 67-year-old male underwent minimally invasive Ivor Lewis esophagectomy on Day 0, with postoperative confirmation of midesophageal squamous cell carcinoma (T_1b_N_1_M_0_). Follow-up imaging 1 week postsurgery showed no complications (Fig. 2). The patient resumed oral intake without discomfort and was discharged on Day 54.

### Readmission due to pulmonary infection

**Figure 3:**
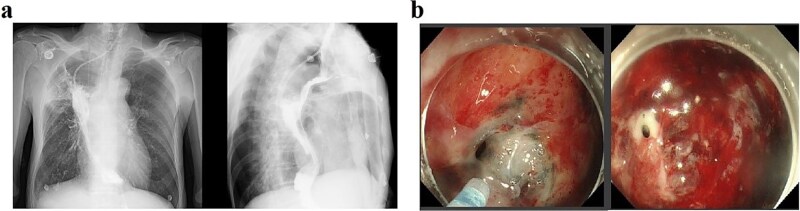
(a) Upper gastrointestinal series obtained 2 months postoperatively. (b) Gastroscopic examination performed 2 months postoperatively.

The patient received two cycles of albumin-bound paclitaxel and cisplatin chemotherapy on Day 52 and Day 76. Following the second cycle, he developed recurrent high-grade fever with productive cough. Laboratory tests revealed elevated inflammatory markers, and chest CT demonstrated severe pulmonary infection unresponsive to antibiotic therapy. Subsequent upper gastrointestinal imaging identified an esophagogastrobronchial fistula (Fig. 3a). Endoscopy revealed a 2 cm necrotic gastric wall defect distal to the anastomosis, accompanied by thickened visceral pleura and a fistulous tract connecting the right lung to the gastric lumen (Fig. 3b). Given the extensive defect and absence of intrathoracic infection, conservative measures, including fasting, gastric decompression, and nutritional support, were initiated.

### Further management

**Figure 4:**
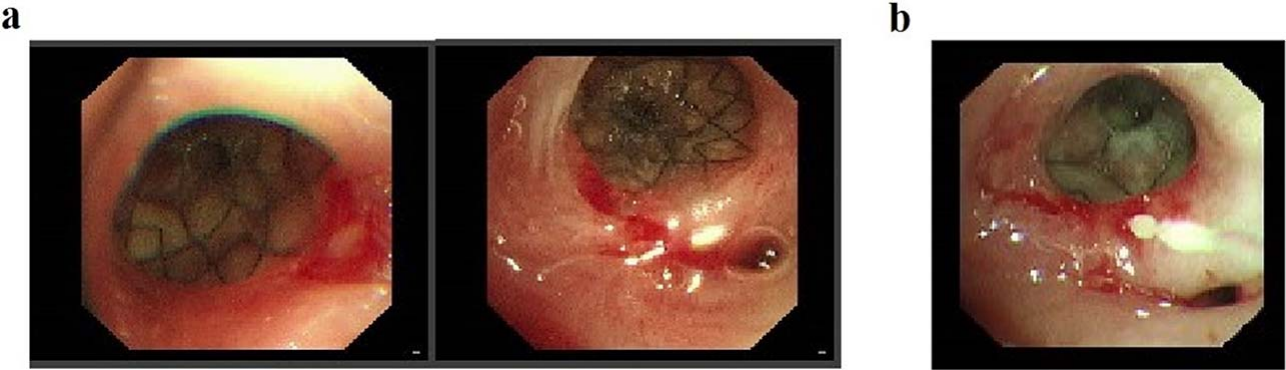
(a) Status following bronchoscopic stent placement. (b) Follow-up bronchoscopy demonstrating healed fistulous tract 2 weeks postintervention.

Following successful control of the pulmonary infection with conservative therapy, two bronchial stents were placed via fiberoptic bronchoscopy in the right mainstem bronchus to isolate the fistulous tract and prevent recurrent infection. Follow-up bronchoscopy after 2 weeks confirmed complete closure of the tract (Fig. 4), with resolution of infection markers and normalization of laboratory parameters. Fasting and gastric decompression were maintained to facilitate pleural tissue repair. After observing adequate thickening of the visceral pleura, oral intake was cautiously reintroduced, progressing from liquids to semisolid foods over 3 months. The patient tolerated this transition without infection recurrence, with sustained normalization of clinical and laboratory findings.

## Discussion

Anastomotic leakage represents one of the most critical complications following esophageal cancer surgery, significantly increasing mortality risk [3]. Early detection and multidisciplinary management are essential to mitigate adverse outcomes [4]. Approximately 80% of postesophagectomy leaks occur during the early postoperative period. Surgical risk factors include improper anastomosis technique (e.g. too tight or too loose suturing) and inadequate intraoperative gastric tissue preservation [5]. Patient-related contributors involve malnutrition, advanced age, obesity, smoking, and comorbidities such as diabetes or preexisting pulmonary conditions [4]. Late-onset leaks are less common, and their etiology remains poorly understood. One plausible explanation is that anastomotic leakage occurred early after the surgery. However, it is difficult to detect in the early stage for the fistula is localized, and the clinical manifestations are not obvious.

Lerut *et al.* classify esophagogastric anastomotic leaks into four clinical categories—type I: radiologically detected leaks without symptoms, requiring no intervention; type II: mild symptoms (e.g. localized cervical wound infection, fever, leukocytosis, elevated CRP) managed with drainage, delayed oral intake, and antibiotics; type III: severe sepsis with endoscopic evidence of anastomotic disruption, necessitating CT-guided drainage; and type IV: endoscopic confirmation of anastomotic necrosis, mandating surgical revision. Leakage of digestive fluids risks systemic infection, though well-contained fistulas often resolve with adequate drainage. Thus, fistula closure and infection control form the cornerstone of management [6]. Current closure techniques include endoscopic clipping [effective for small fistulas (≤5 mm) but limited by fibrosis-induced failure] [7]; endoscopic vacuum therapy (EVAC) (promotes granulation tissue via negative pressure, often requiring repeated applications) [8]; stent implantation combined with fibrin sealant (the combined use can increase the closure rate, especially effective for complex fistulas).

This case report describes a unique presentation of a large esophagogastric anastomotic fistula detected 2 months postoperatively, complicated by severe pulmonary infection. Remarkably, dense adhesions between the right visceral pleura and the intrathoracic gastric conduit localized the infection, preventing systemic dissemination. Chronic inflammatory stimulation induced progressive pleural thickening, resulting in acid-resistant scar tissue that maintained structural integrity despite gastric acid exposure. This adaptive mechanism enabled oral intake, without infection recurrence. Ongoing surveillance with endoscopy and chest CT remains critical to monitor gastric and thoracic cavity status. Resuming fasting and gastric decompression when necessary may be required to facilitate pleural repair during follow-up. The long-term acid resistance of visceral pleura and its protective role warrant further investigation. We will continue to monitor this patient and refine management strategies based on clinical evolution.

In conclusion, this report documents a rare case of late-onset esophagogastric anastomotic fistula where hypertrophied visceral pleura functionally compensated for gastric wall defects, preventing intrathoracic infection spread. While early outcomes appear favorable, long-term follow-up remains essential to validate this compensatory mechanism. Current management of anastomotic fistulas should adhere to established protocols while maintaining flexibility for individualized approaches and choose the appropriate treatment method according to different situations.
